# dSPIC: a deep SPECT image classification network for automated multi-disease, multi-lesion diagnosis

**DOI:** 10.1186/s12880-021-00653-w

**Published:** 2021-08-11

**Authors:** Qiang Lin, Chuangui Cao, Tongtong Li, Zhengxing Man, Yongchun Cao, Haijun Wang

**Affiliations:** 1 School of Mathematics and Computer Science, Northwest Minzu University, Lanzhou, 730030 China; 2 Key Lab of Streaming Data Computing and Applications, Northwest Minzu University, Lanzhou, 730030 China; 3 Department of Nuclear Medicine, Gansu Provincial Hospital, Lanzhou, 730000 China

**Keywords:** SPECT bone scintigraphy, Automated diagnosis, Image classification, Deep learning, CNN

## Abstract

**Background:**

Functional imaging especially the SPECT bone scintigraphy has been accepted as the effective clinical tool for diagnosis, treatment, evaluation, and prevention of various diseases including metastasis. However, SPECT imaging is brightly characterized by poor resolution, low signal-to-noise ratio, as well as the high sensitivity and low specificity because of the visually similar characteristics of lesions between diseases on imaging findings.

**Methods:**

Focusing on the automated diagnosis of diseases with whole-body SPECT scintigraphic images, in this work, a self-defined convolutional neural network is developed to survey the presence or absence of diseases of concern. The data preprocessing mainly including data augmentation is first conducted to cope with the problem of limited samples of SPECT images by applying the geometric transformation operations and generative adversarial network techniques on the original SPECT imaging data. An end-to-end deep SPECT image classification network named dSPIC is developed to extract the optimal features from images and then to classify these images into classes, including metastasis, arthritis, and normal, where there may be multiple diseases existing in a single image.

**Results:**

A group of real-world data of whole-body SPECT images is used to evaluate the self-defined network, obtaining a best (worst) value of 0.7747 (0.6910), 0.7883 (0.7407), 0.7863 (0.6956), 0.8820 (0.8273) and 0.7860 (0.7230) for accuracy, precision, sensitivity, specificity, and F-1 score, respectively, on the testing samples from the original and augmented datasets.

**Conclusions:**

The prominent classification performance in contrast to other related deep classifiers including the classical AlexNet network demonstrates that the built deep network dSPIC is workable and promising for the multi-disease, multi-lesion classification task of whole-body SPECT bone scintigraphy images.

## Background

Malignant tumors characterized by high morbidity and mortality are the major threat to human health. The oncological patients would develop bone metastasis when the solid tumors have invaded to the bone. Clinical statistical findings show that over 60% of metastases originate from breast cancer [1, 2] and the rest stemming from thyroid, lung, and kidney cancers [3]. Generally, a patient suffered from bone metastasis will experience fractures and pain, bringing significant effects on survival time and quality of life. The early detection of bone metastases becomes thus crucial for reasonable choice of treatment strategy and increasing survivability.

The most commonly used clinical tool for surveying the presence or absence of bone metastasis is functional imaging. In contrast to the conventional structural imaging modalities (e.g., CT, MRI and Ultrasound), which capture only the morphological/structural changes, the functional imaging techniques can reveal not only the morphological but also the functional variants in organs, tissues, and parts of the body by acquiring metabolism of radiopharmaceutical in the nuclear medicine domain [4]. Currently, the main techniques of nuclear medicine functional imaging include the single photon emission computed tomography (SPECT) and positron emission tomography (PET). Although PET enables better resolution, SPECT is a more affordable and widely used tool due to the low-cost equipment and radiopharmaceuticals (i.e., 99mTc-MDP). SPECT has been accepted as an effective tool for initial diagnosis of bone and joint changes in patients with oncologic diseases since the early 1990s [5, 6]. More than 18 million SPECT scans are conducted each year in the United States [7].

The SPECT imaging is acquired after 3 h following intravenous injection of radiopharmaceutical (99mTc-MDP), enabling two images of the anterior and posterior views of the body. The SPECT image is stored in a DICOM file as a 2D matrix. The value of elements in the matrix indicates the intensity of radiopharmaceutical uptake and is represented by a 16-bit unsigned integer. Those occupying lesions like bone metastasis are seen as areas of increased radioactivity in SPECT imaging.

SPECT imaging is characterized by low signal-to-noise ratio and inferior spatial resolution, with the size of a whole-body SPECT image being 256 × 1024. Manual analysis of SPECT imaging findings by physicians is very time-consuming, laborious, and subjective. Automated analysis of SPECT images for accurate diagnosis of diseases becomes extremely desired. In the field of traditional machine learning, early studies [8, 9] utilized artificial neural networks to classify features of hot spots that were segmented from bone scintigraphy images. CADBOSS [10] is a computer-aided diagnosis system using image gridding to extract feature of the metastatic regions. The extracted features were further classified with an artificial neural network classifier to determine whether metastases are present or absent. Mac et al. [11] used *k*-nearest neighbor and support vector machine classifiers to identify objects that were extracted from the bone scintigraphy images using edge segmentation method together with the full lambda-schedule algorithm. The parallelepiped classification was applied in [12] to map the radionuclide distribution in scintigraphic images, enabling to detect bone metastasis. The common limitation of these studies is that they identify diseases through classifying the manually extracted features of hot spots. However, manual features extracted by human researchers often suffer from insufficient capability and unsatisfied performance for clinical tasks [13].

Deep learning techniques especially the convolutional neural networks (CNNs) have gained huge success in computer vision due to their ability of automatically extracting hierarchical features from images in an optimal way. CNNs-based image classification and object segmentation become ubiquitous in medical image analysis in recent years [14–16]. With bone scintigraphy images, a large number of CNNs-based works have been done for automated diagnosis of bone metastasis. The master’s thesis [17] from Lund University is the earliest work on identifying whether the hotspots in bone scintigraphy images represent bone metastases caused by prostate cancer or other physiological process. A five-branch CNN model has been developed to classify image patches of already found hotspots in the spine, with each branch corresponding an image patch. The trained model has reached an accuracy of 89% on the testing dataset. A CNN-based model consisting of three sub-networks (i.e., feature extraction, aggregation and classification) was proposed in [18] to determine the absence or presence of bone metastasis. The model was evaluated using 1600 samples of bone scintigraphy images, demonstrating an accuracy of 95% with two-view inputs (i.e., the anterior and posterior image). Using the similar three-stage network in [18], Zhao et al. [19] studied to classify bone scintigraphy images for identifying bone metastasis caused by various solid tumors, achieving an AUC value of 98.8% for breast cancer, 95.5% for prostate cancer, 95.7% for lung cancer, and 97.1% for other cancers on a testing dataset consisting of 1223 cases. Papandrianos et al. [20–23] studied to classify bone scintigraphy images for diagnosis of bone metastasis caused by prostate cancer [20–22] and breast cancer [23] with simple CNN-based models. Their models achieved a highest two-class classification accuracy of 97.38% for prostate cancer and an accuracy of 92.5% for breast cancer. Cheng et al. [24, 25] used YOLO models [26, 27] to detect lesions of chest and pelvis bone metastasis in scintigraphic images from prostate and breast cancer patients. Their developed CNNs-based classification models achieved a mean precision of 90% for classifying the detected lesions in the chest [26], and a precision of 70% (81%) for classifying the detected lesions in the chest (pelvis) [27]. In our previous work [28], we developed a group of CNNs-based two-class classification models to identify bone metastasis in the thoracic images clipped from whole-body SPECT images, obtaining a best accuracy of 98.7% on the testing samples of augmented dataset.

Existing research efforts mentioned above focus only on the automated classification of scintigraphy images to determine that whether an image contains bone metastasis or not, falling into the line of two-class (binary) classification problem. However, it is not rare that different diseases may be present in a single scintigraphy image because the whole-body SPECT imaging can show the whole skeletal structure of a patient. Moreover, there may be confusion in the manifestation of bone metastasis and those non-oncological diseases including arthropathies.

To automatically detect different diseases in whole-body SPECT scintigraphy images, in this work, we self-define a CNN-based classification network that is able to determine whether and what diseases being contained in given images, by classifying these images into classes (i.e., the normal, metastatic, arthritic, and metastatic & arthritic). Specially, different data augmentation methods are used to extend the dataset for coping with the problem of limited samples of SPECT images. CNN-based network dSPIC is developed to first extract optimal features from whole-body SPECT images and then classify these images into classes of concern. A dataset consisting of a group of real-word whole-body SPECT images is constructed to evaluate the self-defined end-to-end classification network.

The main contributions of this work can be concluded as follows

- First, we identify the research problem of automated multi-disease, multi-lesion diagnosis with whole-body SPECT images and convert the problem into multi-class classification of scintigraphic images. To the best of our knowledge, this is the first work in the field of multi-class classification of SPECT images. By contrast, existing research efforts including our previous work in [28] were conducted for two-class classification of whole-body or partial SPECT scintigraphic images.
- Second, we develop a self-defined CNN-based classification network that is able to automatically extract hierarchal features of lesions from images in an optimal way and classify the high-level features into classes of concern. This can alleviate, to a great extent, the insufficient capability and unsatisfied performance of handcrafted features extracted by human researchers in the field of conventional machine learning-based image analysis.
- Last, we use a group of clinical scintigraphic images to evaluate the self-defined network. The experimental results show that our method performs well on detecting diseases in whole-body SPECT scintigraphic images in terms of the defined evaluation metrics (i.e., accuracy, precision, sensitivity, specificity, F-1 score, and AUC value).

The rest of this paper is organized as follows. The used data of SPECT images and the self-defined network will be detailed in Sect. 2. Experimental evaluation conducted on real-world data will be provided in Sect. 3. A brief discussion will be provided in Sect. 4. And in Sect. 5, we conclude this work and point out the future research directions.

## Methods

In this section, the used dataset of whole-body SPECT bone scans is outlined, followed by a description of the data pre-processing and the developed deep classification network.

### Dataset

The whole-body SPECT images used in this work were collected from the Department of Nuclear Medicine, Gansu Provincial Hospital in 2018. In the process of examination, patients were intravenously injected with radionuclides 99mTc-MDP (740 MBq), which were then acquired after about three hours by using a Siemens SPECT ECAM imaging equipment outside the body of the patients.

**Fig. 1:**
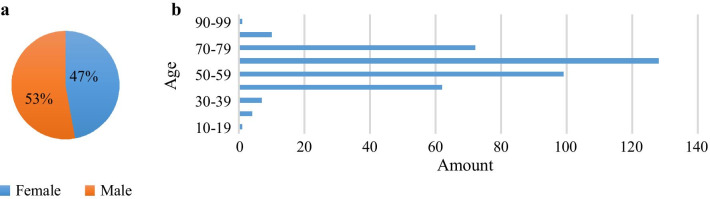
Distribution of patients included in the dataset of whole-body SPECT images. **a** Gender; and **b** Age

Patients with bone metastasis, arthritis or both of them are considered in this study, consisting of 181 female patients and 203 male patients. Figure 1 provides the distribution of patients with respect to gender and age.

**Fig. 2:**
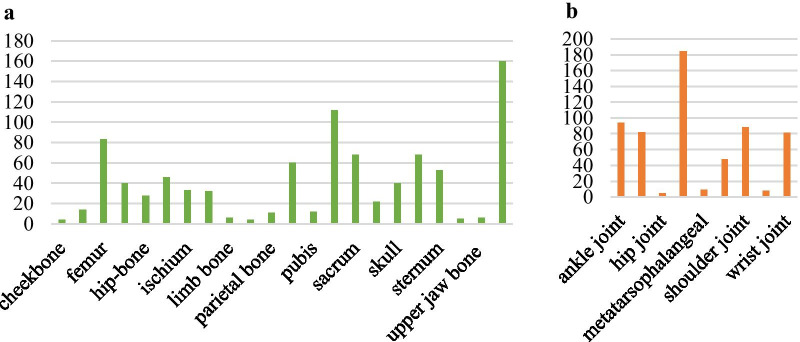
An illustration of lesion distribution in the selected whole-body SPECT images. **a** Bone metastasis with 907 lesions of 182 (174 + 8) images; and **b** Arthritis with 599 lesions of 260 (252 + 8) images

Generally, SPECT imaging process outputs two images (i.e., the anterior- and posterior-view image) for every examination and each image is stored in a DICOM (Digital Imaging and Communications in Medicine) file. The collected 768 whole-body SPECT images from 384 patients fall into four classes of concern, i.e., the normal (n = 334, ≈43.5%), metastatic (n = 174, ≈22.7%), arthritic (n = 252, ≈32.8%), and metastatic & arthritic (n = 8, ≈1.0%). The lesion distribution shown in Fig. 2 reveals that the vertebra, rib, and femur are the top three skeletal areas where bone metastasis frequently occurs and arthritis often presents in knee joint.

We can see from the lesion distribution in Fig. 2 that, in general, there are more than one lesion in an image. Moreover, eight whole-body SPECT images in the metastatic & arthritic class include metastatic and arthritic lesions simultaneously. The objective of this work is to develop a CNN-based classification method for multi-disease, multi-lesion diagnosis with whole-body SPECT images.

### Overview

**Fig. 3:**
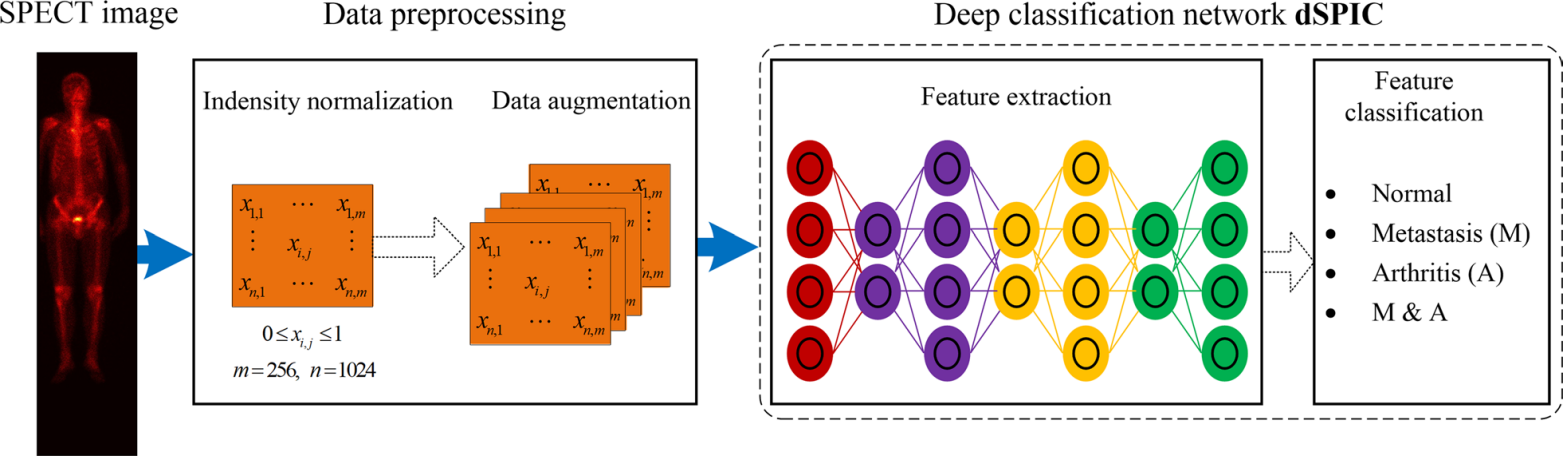
Overview of the proposed CNN-based SPECT image classification method consisting of data preprocessing, feature extraction and feature classification

Figure 3 shows the overall process of automated diagnosis of diseases in whole-body SPECT images by using CNN-based classification network, consisting of two main stages.

- *Stage* 1: Data preprocessing including intensity normalization and data augmentation is utilized to first keep the acquired varying intensity of radiopharmaceuticals within a fixed interval and then generate more samples of SPECT images. This can facilitate the CNN-based model extract more rich features from ‘big data’ of samples.
- *Stage* 2: A self-defined end-to-end classification network, dSPIC, extracts hierarchical features from the augmented data of SPECT images and classify the high-level features into one of the four classes, i.e., the normal (N), metastatic (M), arthritic (A), and metastatic & arthritic (M&A).

In the subsequent sections, the data processing methods and the self-defined classification network, dSPIC, will be elaborated.

### Data preprocessing

**Fig. 4:**
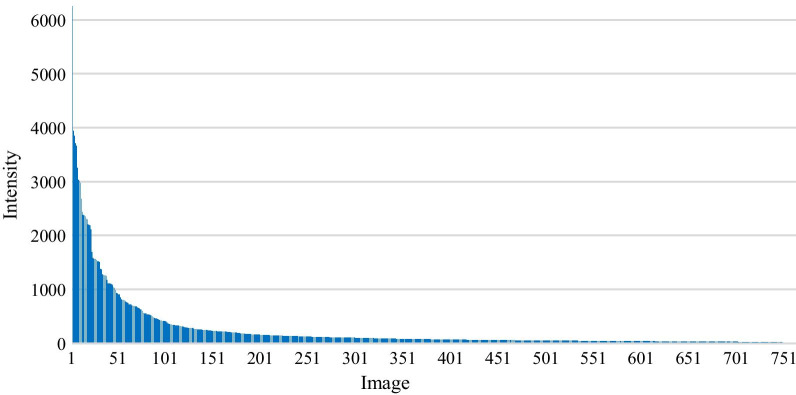
An illustration of the maximum of uptake intensity from all SPECT images in the original dataset

SPECT imaging with 99mTc-MDP often demonstrates intensive radiopharmaceutical uptake in bone with a large-mineralizing surface area (e.g., spine) compared to the shafts of long bones [29]. As illustrated in Fig. 4, the large variability in intensity of radiopharmaceuticals makes SPECT images significantly different from the natural images in which the value of pixel ranges from 0 to 255. To mitigate the effect of the varying intensity on feature extraction and representation, in this work, each DICOM file will be self-adaptively normalized to keep every element within a fixed interval according to its maximum and minimum of intensity.

For *x*_*i*_ (1 ≤ *i* ≤ *m* × *n*) denoting the intensity of the *i*-th element in a SPECT image with size of *m* × *n*, let *x*_max (*x*_min) be the maximum (minimum) of intensity in this image, a normalized value *x*_*N*_ can be calculated using the min–max normalization method as follows.

1 $$x_{N} = \frac{x - x\_\min }{{x\_\max\, - \, x\_\min }}.$$

For the whole-body SPECT images used in this work, *m* = 256 and *n* = 1024. The normalized SPECT images are organized into dataset D_1. The subsequent data augmentation is conducted on the samples in dataset D_1.

It is widely accepted that the classification performance of CNN-based models depends on the size of dataset, with high classification accuracy always corresponding to the large dataset. For that reason, we harvest more samples of images by augmenting dataset D_1 with the parameter variation and sample generation techniques. A concomitant effect of data augmentation is to improve robustness of CNN-based model for coping with the patient-related artifacts during imaging.

#### Data augmentation using parameter variation

For a point (*x*_*i*_, *y*_*i*_) in the given image **X**, we can calculate its corresponding point (*x*_*o*_, *y*_*o*_) in the mirror counterpart **X**_M_ according to Eq. 2.

2 $$\left[ {\begin{array}{*{20}c} {x_{o} } \\ {y_{o} } \\ 1 \\ \end{array} } \right] = \left[ {\begin{array}{*{20}c} { - 1} & 0 & w \\ 0 & 1 & 0 \\ 0 & 0 & 1 \\ \end{array} } \right]\left[ {\begin{array}{*{20}c} {x_{i} } \\ {y_{i} } \\ 1 \\ \end{array} } \right].$$

The outputted points in images **X**_T_ and **X**_R_ obtained via translating **X** by ± *t* pixels in horizontal or vertical direction and rotating **X** by ± *r* degrees in left or right direction can be calculated according to Eqs. 3 and 4.

3 $$\left[ {\begin{array}{*{20}c} {x_{o} } \\ {y_{o} } \\ 1 \\ \end{array} } \right] = \left[ {\begin{array}{*{20}c} { - 1} & 0 & w \\ 0 & 1 & 0 \\ 0 & 0 & 1 \\ \end{array} } \right]\left[ {\begin{array}{*{20}c} {x_{i} } \\ {y_{i} } \\ 1 \\ \end{array} } \right],$$

4 $$\left[ \begin{gathered} x_{o} \hfill \\ y_{o} \hfill \\ \end{gathered} \right] = \left[ {\begin{array}{*{20}c} 1 & 0 \\ 0 & 1 \\ \end{array} } \right]\left[ {\begin{array}{*{20}c} {x_{i} } \\ {y_{i} } \\ \end{array} } \right] \pm \left[ {\begin{array}{*{20}c} {t_{x} } \\ {t_{y} } \\ \end{array} } \right]$$

The values for *t* and *r* mentioned above are experimentally determined in this work. Now, we obtain a new augmented dataset D_2 that is outlined in Table 1. We can see from Table 1 that all diseased classes have been augmented while the normal class keeps unchanged.

**Table 1:** The augmented dataset D_2

	N	M	A	M&A
Samples	334	520	568	80
Ratio (%)	22.3	34.6	37.8	5.3

*N* normal,* M* metastatic,* A* arthritic,* M&A* metastatic and arthritic

#### Data augmentation using sample generation

Generative adversarial network (GAN) [30] as one of the most emerging deep learning techniques can be used to generate new samples with the given images. The generated samples have entirely different distribution from the original ones. Deep convolutional generative adversarial network (DCGAN) [31] is the recent innovation of GAN. We apply DCGAN to generate samples with images in dataset D_1 and organize these generated samples in dataset D_3 (see Table 2).

**Table 2:** The augmented dataset D_3

	N	M	A	M&A
Samples	334	394	472	80
Ratio (%)	26.1	30.8	36.9	6.2

*N* normal,* M*  metastatic,* a* arthritic,* M&A * metastatic and arthritic

### Supervised classification network dSPIC

In this work, we self-define a deep SPECT Image Classification network (dSPIC) for automated diagnosis of diseases of concern. Table 3 outlines the network architecture of dSPIC, consisting of seven weight layers (i.e., the convolutional and fully connected), one added layer, and one Softmax layer.

**Table 3:** The architecture of the self-defined dSPIC network

Layer	Configuration
Convolution	11 × 11, 96, S = 4, P = 2
Pooling	MaxPool, 3 × 3, S = 2
Added layer	Attention/residual module
Convolution	5 × 5, 256, S = 1, P = 2
Pooling	MaxPool, 3 × 3, S = 2
Convolution	3 × 3, 384, S = 1, P = 3
Convolution	3 × 3, 384, S = 1, P = 3
Convolution	3 × 3, 256, S = 1, P = 1
Fully connected	4096
Fully connected	4096
SoftMax	3

*S* stride,* P* padding,* MaxPool* max pooling

#### Convolutional layer

This layer uses a linear filter to produce the feature maps. A total of five convolutional layers are included in dSPIC, denoting as < kernel_size, channel_number, stride_size, padding_size >. The size of convolutional kernel and stride keeps on decreasing while the number of channel increasing except from the one of the last convolution layer. Every convolutional layer has a group of filters with different kernel_size. In the first convolutional layer, the input 256 × 1024 SPECT image is convolved with each filter of 11 × 11 to calculate a feature map made of neurons, which is followed by a pooling layer. Since the local connectivity of convolutional operation, dSPIC can learn filters that maximally respond to a local region (e.g., lesions) of the input, thus exploiting the spatial local correlation of the input [32]. Similarly, the subsequent convolutional layers take the feature maps of immediately previous layers as inputs to convolve with each filter.

#### Pooling layer

This layer completes the downsampling operation that is typically applied after a convolution layer. Max and average pooling are special kinds of pooling where the maximum and average value is taken, respectively. The similar information from the neighborhood of a receptive field covered by a filter will be captured through outputting a dominant response within this local region, enabling the input be invariant to geometrical distortions. Specifically, the max pooling is used in dSPIC to partition an input image or feature map into a set of sub-regions with size of 3 × 3, and output the maximum value for each such sub-region.

#### The added layer

**Fig. 5:**
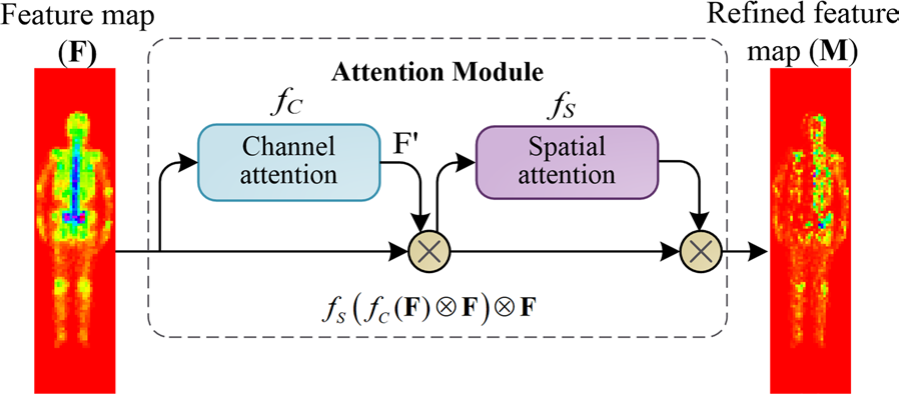
The attention module used in dSPIC, consisting of a channel sub-module and a spatial attention sub-module

We introduce attention mechanism and residual module [33] into the network to improve dSPIC focusing on those more important areas (i.e., lesions) on the feature maps by considering the important information, or reducing the training parameters and time. As depicted in Fig. 5, the attention module consists of two sequential sub-modules, i.e., the channel attention module and spatial attention module.

The channel attention module in Fig. 5 produces a 1D output F′ for an input of 2D feature map **F**. The vector F′ will be fed into the spatial attention module to obtain a refined 2D feature map **M**. Formally, **M** is calculated according to Eq. 6.

6 $${\mathbf{M}} = f_{S} \left( {f_{C} ({\mathbf{F}}) \otimes {\mathbf{F}}} \right) \otimes {\mathbf{F}},$$

where $\otimes$ is the element-wise multiplication; and *f*_*C*_ and *f*_*S*_ is the channel and spatial function, respectively.

In detail, the channel attention F′ = *f*_*C*_ and spatial attention **M** = *f*_*S*_ are calculated according to Eqs. 7 and 8.

7 $$f_{C} ({\mathbf{F}}) = \sigma \left( {MLP\left( {AvgPool({\mathbf{F}})} \right) + MLP\left( {MaxPool({\mathbf{F}})} \right)} \right),$$

8 $$f_{S} ({\mathbf{F}}^{\prime } ) = \sigma \left( {f^{7 \times 7} \left( {\left[ {AvgPool({\mathbf{F}}^{\prime } );MaxPool({\mathbf{F}}^{\prime } )} \right]} \right)} \right),$$

where *σ* denotes the sigmoid function, *MLP* is the multi-layer perceptron, AvgPool (MaxPool) represents the average (max) pooling, and *f* ^7×7^ is a convolutional operation with the kernel size of 7 × 7.

**Fig. 6:**
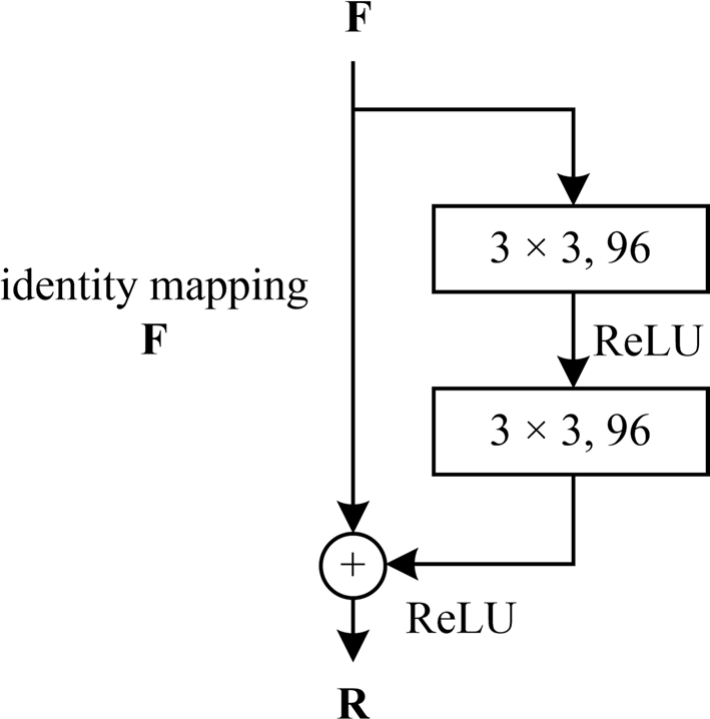
The residual module used in dSPIC

The introduced residual modules is shown in Fig. 6 where two 3 × 3 convolutional layers and a ReLU layer after the first convolutional layer are added.

Given an input of 2D feature map **F**, the residual module will output a 2D output **R**, which is mathematically represented as follows.

9 $${\mathbf{R}} = \delta \left( {f^{3 \times 3} \left( {\delta \left( {f^{3 \times 3} ({\mathbf{F}})} \right)} \right) + {\mathbf{F}}} \right),$$

where *δ* is the ReLU function, and *f* ^3 × 3^ is a convolutional operation with the kernel size of 3 × 3.

The skip connection indicating by the identify mapping path in Fig. 6 enables the same output with the input, i.e., **R** = **F**.

#### Fully connected layer

A fully connected layer has a set of full connections to all activations in its previous layer. The activations can be computed with a matrix multiplication followed by a bias offset. There are two fully connected layers in dSPIC network to make non-linear combination of the selected features at the end of the network.

#### Softmax layer

The network output nodes use the Softmax function for the number of the unordered classes. For the case that an image contains the metastatic and arthritic lesions simultaneously, the outputs of the top-1 and -2 probability indicate the classes, respectively. A Softmax function is defined in Eq. 10.

10 $$f(x_{j} ) = \frac{{e^{{x_{j} }} }}{{\sum\nolimits_{i = 1}^{n} {e^{{x_{i} }} } }},$$

where *f* (*x*_*j*_) is the score of the *j*-th output node, *x*_*j*_ is the network input to *j*-th output node, and *n* is the number of output nodes. In fact, all of the output values *f* (*x*) are a probability between 0 and 1, and their sum is 1.

Furthermore, the nonlinear function used in dSPIC network is the ReLU (rectified liner unit) function, which enables dSPIC to approximate arbitrarily complex functions. The input of a non-linear processing layer is the output of its immediate previous convolution layer. For a given input *x*, ReLU is mathematically defined as follows.

11 $${\text{ReLU}}(x) = \max (0,x)$$

The used optimizer in dSPIC is Adam (adaptive moment estimation) [34], which has been proved to be well suited for the problems with large-size data and parameters like whole-body SPECT images. Adam typically performs smaller updates for the frequent parameters and larger updates for the infrequent parameters. Let *θ*_*t*_ denote the parameter vector at timestep *t*, Eq. 12 provides the Adam’s update rule [34].

12 $$\theta_{{t + {1}}} = \theta_{t} - \alpha \frac{{\vec{m}_{t} }}{{\sqrt {\vec{v}_{t} } + \varepsilon }}\left| \begin{gathered} \vec{m}_{t} = \frac{{m_{t} }}{{1 - \beta_{1}^{t} }} \hfill \\ \vec{v} = \frac{{v_{t} }}{{1 - \beta_{2}^{t} }} \hfill \\ \end{gathered} \right.,$$

where *α* is the stepsize, *ε* is a constant; *m*_*t*_ = *β*_1_*m*_*t*–1_ + (1–*β*_1_)∙*g*_*t*_ denotes the biased first moment estimate, and *v*_*t*_ = *β*_2_*v*_*t*–1_ + (1–*β*_2_)∙$\beta_{2}^{t}$ represents the biased second raw moment estimate. *g*_*t*_ ← ∇*θf*_*t*_(*θ*_*t*−1_) denotes the gradients with respect to stochastic objective at timestep *t*; and *β*_1_, *β*_2_ ∈ [0, 1) are the exponential decay rates for the moment estimates.

To examine the effect of attention module and residual module on classification performance of dSPIC, two classifiers dSPIC-AM (attention module) and dSPIC-RM (residual module) will be evaluated separately in the experimental validation section below.

## Results

In this section, we provide an experimental evaluation of the self-defined dSPIC network using a set of real-world whole-body SPECT images, which have been organized into three different datasets, i.e., D_1 (original dataset without augmentation), D_2 (augmented dataset using parametric variation), and D_3 (augmented dataset with CGAN). This section begins with an illustration of the SPECT image annotation.

### SPECT image annotation

Labelling SPECT image to obtain ground truth plays a crucial role for training a reliable supervised classifier. However, it is a time-consuming and laborious task due to the inferior spatial resolution of SPECT imaging. In this work, we develop an annotation system based on the openly available online tool LabelMe released by MIT (http://labelme.csail.mit.edu/Release3.0/), to facilitate manual annotation by nuclear medicine physicians as far as possible.

**Fig. 7:**
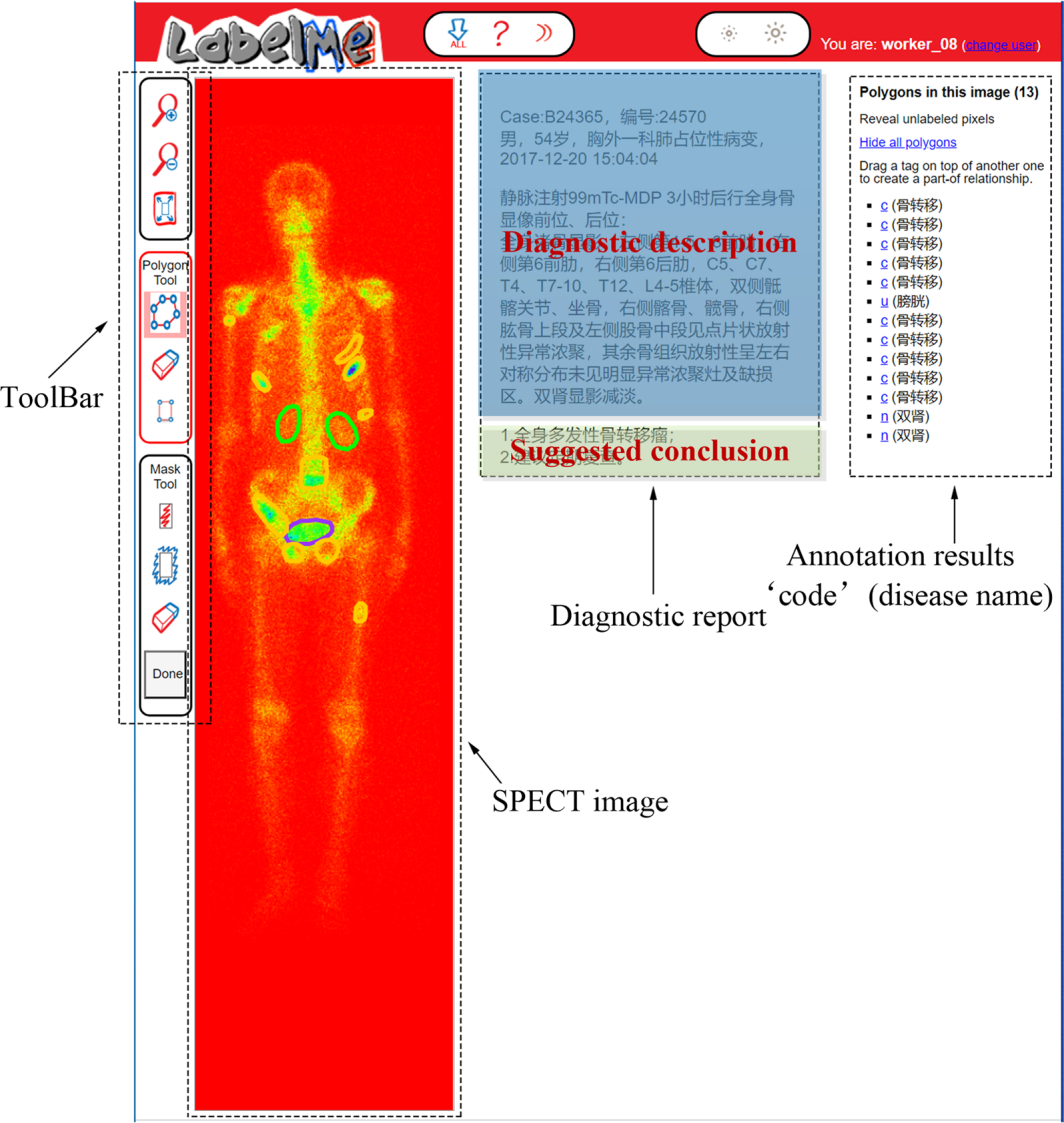
An illustration of labelling SPECT image by using the developed LabelMe annotation system

With LabelMe-based annotation system, SPECT imaging findings including the DICOM file and the textual diagnostic report can be imported into the system in advance (see Fig. 7). In the labelling process, three nuclear medicine physicians from Department of Nuclear Medicine, Gansu Provincial Hospital who are members of our research group were asked to manually label areas on the visual presentation of DICOM file (RGB format currently used but not limited to this) with a shape tool (e.g., polygon and rectangle) in the toolbar. The labelled area will be annotated with a self-defined code combined with the name of disease or body part. The results of manual annotation for all SPECT images serve as ground truth in the experiments and form an annotation file together to feed into the classifiers.

The image annotation process was performed by physicians independently according to the diagnosis report consisting of diagnostic description and suggested solution (see Fig. 7). If the majority of them (i.e., at least two of them) think that an image is abnormal (i.e., at least one lesion presents in it), it is labeled as a positive one; otherwise, it is labelled as a negative image. A labeled abnormal SPECT image belongs to one of the disease classes, i.e., the metastatic, arthritic, and metastatic & arthritic.

### Experimental setup

The evaluation metrics we use are *accuracy*, *precision*, *recall*, *specificity*, *F*-1 score and AUC (Area Under ROC Curve). In practice, a classified SPECT image falls into one of the four categories:

- True Positive (*TP*), which correctly identifies an abnormal image as positive;
- False Positive (*FP*), which incorrectly identifies a normal image as positive;
- False Negative (*FN*), which incorrectly identifies an abnormal image as normal; and
- True Negative (*TN*), which correctly identifies a normal image as normal.

Accordingly, we define accuracy, precision, sensitivity, specificity, and F-1 score in Eqs. 13–17.

13 $$Accuracy = \frac{TP + TN}{{TP + TN + FP + FN}},$$

14 $$Precision = \frac{TP}{{TP + FP}},$$

15 $$Sensitivity = Recall = \frac{TP}{{TP + FN}},$$

16 $$Specificity = \frac{TN}{{TN + FP}},$$

17 $$F - 1 = 2 \times \frac{Precision \times Sensitivity}{{Precision + Sensitivity}}$$

It is desirable that a classifier should have both a high true positive rate (*TPR* = Sensitivity), and a low false positive rate (*FPR* = 1–Specificity) simultaneously. The ROC curve shows the true positive rate (*y*-axis) against the false positive rate (*x*-axis), and the AUC value is defined as the area under the ROC curve. As a statistical explanation, the AUC value is equal to the probability that a randomly chosen positive image is ranked higher than a randomly chosen negative image. Therefore, the closer to 1 the AUC value is, the higher performance the classifier achieves.

We divide every dataset (D_1, D_2 and D_3) into parts: training set and testing set, with the ratio of them is 7: 3. It means that we use 70% of samples in each dataset to train the classifiers, and the rest 30% for testing the classifiers. The parameters setting can been seen in Table 4.

**Table 4:** Parameters setting of the multi-class, multi-lesion deep classification network dSPIC

Parameter	Value
Learning rate	0.0001
Optimizer	Adam
Batch size	8/16
Epoch	300

The experiments are run in Tensorflow 2.0 on an Intel Core i7-9700 PC with 32 GB RAM running Windows 10.

### Experimental results

In this section, we first examine the impacts of the size of the dataset and data augmentation on classification performance by providing quantitative results of evaluation metrics obtained by dSPIC-AM and dSPIC-RM. Then the classifier with the highest performance will be used to provide a comparative analysis between some classical CNN-based classification networks with the same dataset.

Tables 5 and 6 present the average quantitative values of the evaluation metrics obtained by dSPIC-AM and dSPIC-RM, respectively. On the whole, dSPIC-AM outperforms dSPIC-RM on classifying SPECT images in terms of the defined evaluation metrics. Data augmentation positively contributes to improving classification performance, with an increase of 8.37% (6.30%) for accuracy (F-1 score) metric by the classifier dSPIC-AM. Parametric variation is more suitable for augmenting samples with the current dataset of SPECT images than the DCGAN-based sample generation technique.

**Table 5:** Average quantitative values of evaluation metrics obtained by the classifier dSPIC-AM

Dataset	Accuracy	Precision	Sensitivity	Specificity	F-1 score
D_1	0.6910	0.7412	0.6956	0.8273	0.7230
D*_*2	0.7747	0.7883	0.7863	0.8820	0.7860
D*_*3	0.7286	0.7407	0.7528	0.8514	0.7287

**Table 6:** Average quantitative values of evaluation metrics obtained by the classifier dSPIC-RM

Dataset	Accuracy	Precision	Sensitivity	Specificity	F-1 score
D*_*1	0.6955	0.7324	0.6978	0.8345	0.7147
D*_*2	0.7558	0.7437	0.7566	0.8650	0.7530
D*_*3	0.7389	0.7606	0.7756	0.8543	0.7680

**Fig. 8:**
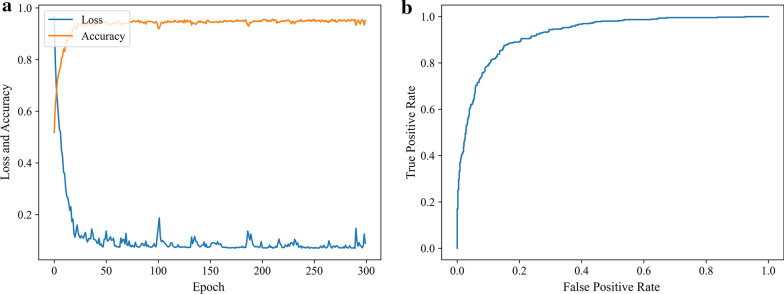
The training and testing performance of dSPIC-AM on dataset D_2. **a** Accuracy and loss curves obtained by training classifier; and **b** ROC curve obtained by testing classifier with a value of 0.9272 for AUC

We can conclude that dSPIC-AM is the highest-performance classifier with the best values of all evaluation metrics conduced on testing samples in dataset D_2. The strength of dSPIC-AM can be further demonstrated by the training and testing performance in Fig. 8.

**Fig. 9:**
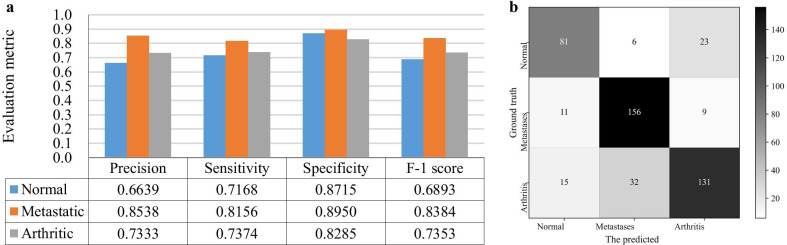
Performance of dSPIC-AM on identifying SPECT images in classes of concern with testing samples in dataset D_2. **a** Individual values for all evaluation metrics; and **b** Confusion matrix

We further show the ability of dSPIC-AM on identifying SPECT images in classes of concern by providing the individual values for all evaluation metrics and the confusion matrix conducted on the testing samples in dataset D_2 in Fig. 9.

To provide a comparative analysis between dSPIC-AM and other deep networks, we define a group of CNN-based classification networks including the classical AlexNet network [35]. The corresponding classifiers have the similar structures but different network depth and parameters from dSPIC-AM (see Table 7).

**Table 7:** Structures of the deep networks used for comparative analysis. (MaxPool(3) = 3 × 3 pooling layer)

SI_CLF	SI_CLF + AM	SI_CLF + RM	SI_CLF + AM + RM	AlexNet
11 × 11, 64, S = 4, P = 2	11 × 11, 64, S = 4, P = 2	11 × 11, 64, S = 4, P = 2	11 × 11, 64, S = 4, P = 2	11 × 11, 64, S = 4, P = 2
MaxPool(3), S = 2	MaxPool(3), S = 2	MaxPool(3), S = 2	MaxPool(3), S = 2	MaxPool(3), S = 2
7 × 7, 128, S = 1, P = 2	AM	RM	RM	5 × 5, 256, S = 1, P = 2
MaxPool(3), S = 2	7 × 7, 128, S = 1, P = 2	7 × 7, 128, S = 1, P = 2	AM	MaxPool(3), S = 2
5 × 5, 128, S = 1, P = 3	MaxPool(3), S = 2	MaxPool(3), S = 2	7 × 7, 128, S = 1, P = 2	3 × 3, 384, S = 1, P = 3
3 × 3, 256, S = 1, P = 3	5 × 5, 128, S = 1, P = 3	5 × 5, 128, S = 1, P = 3	MaxPool(3), S = 2	3 × 3, 384, S = 1, P = 3
3 × 3, 256, S = 1, P = 1	3 × 3, 256, S = 1, P = 3	3 × 3, 256, S = 1, P = 3	5 × 5, 128, S = 1, P = 3	FC (1024)
MaxPool(3), S = 2	3 × 3, 256, S = 1, P = 1	3 × 3, 256, S = 1, P = 1	3 × 3, 256, S = 1, P = 3	FC (1024)
FC (1024)	AM	MaxPool(3), S = 2	3 × 3, 256, S = 1, P = 1	Softmax (3)
FC (1024)	MaxPool(3), S = 2	FC (1024)	AM	
Softmax (3)	FC (1024)	FC (1024)	MaxPool(3), S = 2	
	FC (1024)	Softmax (3)	FC (1024)	
	Softmax (3)		FC (1024)	
			Softmax (3)	

**Fig. 10:**
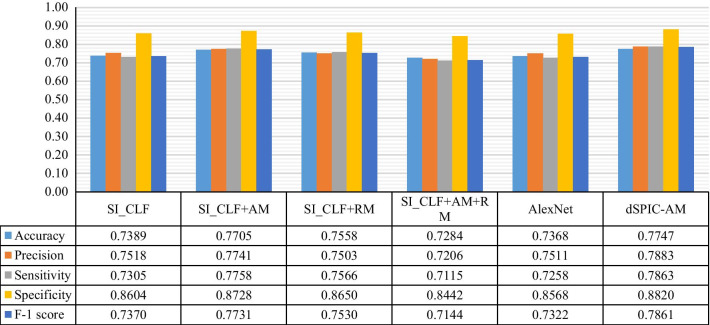
Average quantitative values of evaluation metrics obtained by classifiers in Table 7 and dSPIC on testing samples in dataset D_2

With testing samples in dataset D_2, Fig. 10 provides the average quantitative values of evaluation metrics obtained by classifiers in Table 7 and dSPIC-AM. The corresponding AUC values are presented in Table 8.

**Table 8:** AUC values obtained by classifiers in Table 7 and dSPIC on testing samples in dataset D_2

Classifier	SI_CLF	SI_CLF + AM	SI_CLF + RM	SI_CLF + AM + RM	AlexNet	dSPIC-AM
AUC	0.8904	0.9105	0.9088	0.8859	0.8756	0.9272

**Fig. 11:**
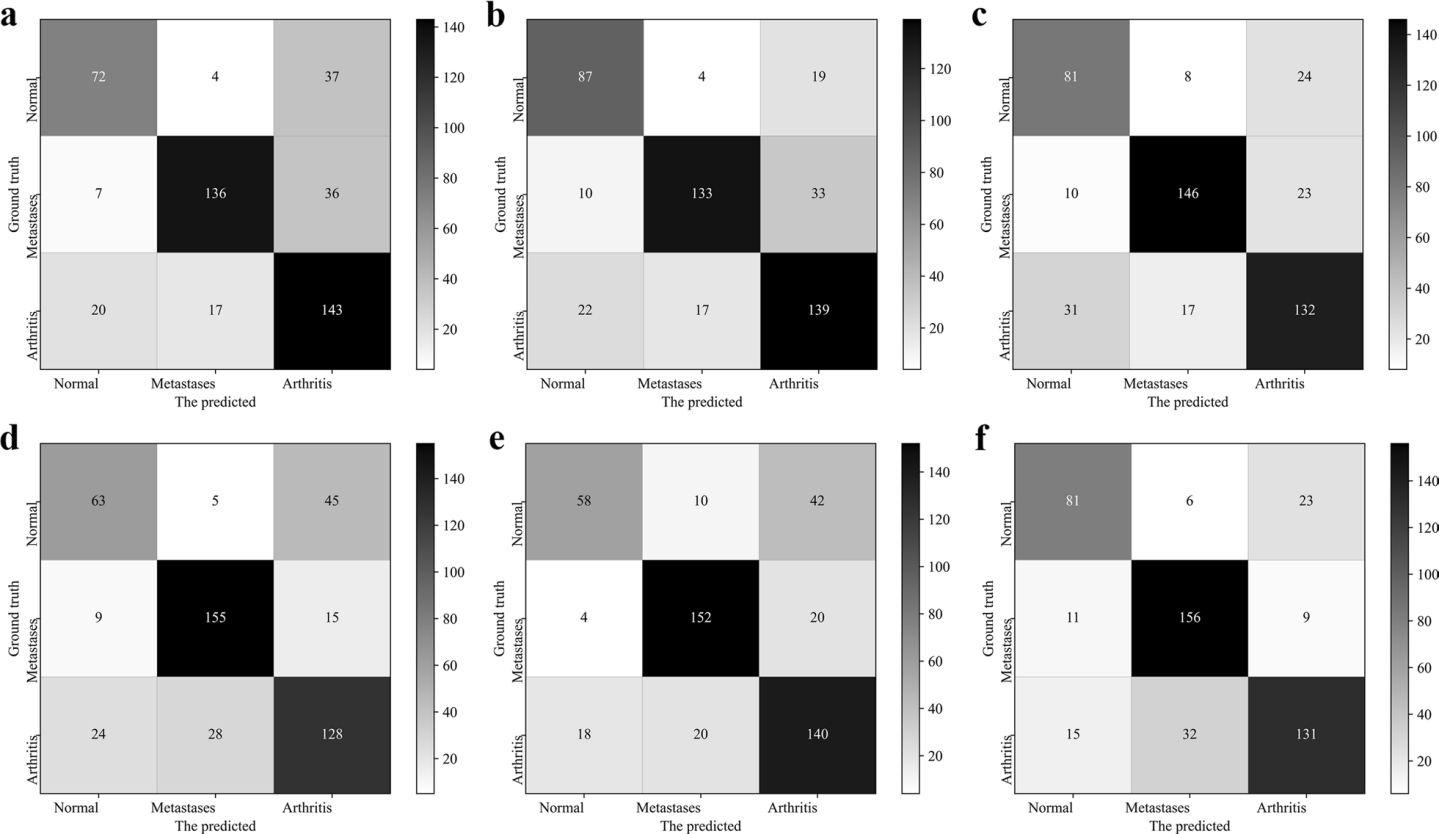
Confusion matrixes obtained by classifiers in Table 7 and dSPIC-AM on testing samples in dataset D_2. **a** SI_CLF; **b** SI_CLF + AM; **c** SI_CLF + RM; **d** SI_CLF + AM + RM; **e** AlexNet; and **f** dSPIC-AM

The confusion matrices obtained by the classifiers in Table 7 and dSPIC-AM are depicted in Fig. 11, further showing the higher performance of dSPIC-AM on classifying SPECT images in different classes.

## Discussion

In this section, we provide a brief discussion about the developed CNN-based network dSPIC on automated multi-disease, multi-lesion classification of whole-body SPECT images from the following aspects.

### Attention module vs. residual module

The channel and spatial attention modules used in this work have the potential to make CNN-based network focusing on the areas of interesting (i.e., lesions) in large-size SPECT images. More representative features of lesions can thus be extracted from limited samples of SPECT images, enabling better performance on classifying high-level features into classes by dSPIC-AM. By contrast, residual operations are often used to reduce training time and eliminate the degradation and gradient vanishing problem frequently faced by deep networks. So, the relatively lower values of evaluation metrics have been achieved by dSPIC-RM. This can be further confirmed by the performance of classifiers in Table 7. As shown in Fig. 9, the classifier SI_CLF + AM outperforms those with residual module and even the classical AlexNet network.

### Impact of dataset size on classification performance

Dataset size is considered a major concern in medical domain [36]. The lack of large-scale dataset brings huge challenge to deep learning based classification models for extracting representative features from images, and hence the inferior classification performance. This is particularly true for our classification problem. The proposed classifiers dSPIC-AM/RM perform better on the augmented datasets (i.e., D_2 and D_3) than the original one (i.e., D_1). Although the huge difficulty of collecting large-scale labeled SPECT images, more samples are still needed for developing high-performance CNN-based classifiers.

### Parametric variation vs. DCGAN-based sample generation

Sample generation was commonly widely used in the field of the deep learning based computer vision, where GAN model and its variant DCGAN are the top choice for generating samples. The generated samples, however, are fake and have different distribution from the original ones. This is why the proposed dSPIC network performs relatively better on dataset D_2 in which the samples augmented by using parametric variation including mirroring, translation, and rotation operations distribute identically.

### Automated classification vs. manual diagnosis

**Fig. 12:**
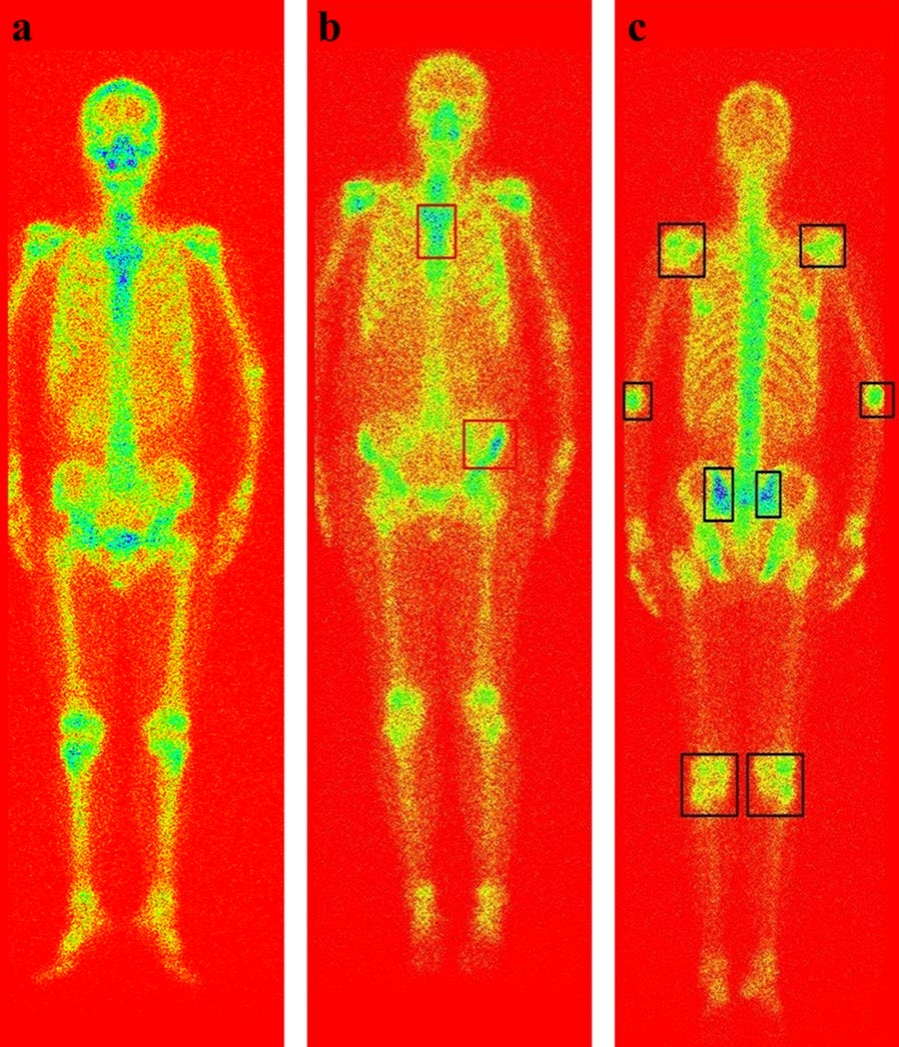
Misclassified examples by dSPIC on testing samples in dataset D_2. **a** The normal identified as arthritis; **b** The metastatic identified as normal; and **c** The arthritic identified as normal
- It is problematic to interpret hotspots (i.e., higher uptake) in the vertebrae/spine since degenerative diseases are often indistinguishable from bone metastases with only SPECT bone imaging. So, the metastatic lesion in thoracic vertebra as depicted in Fig. 12b was incorrectly identified as normal. Moreover, asymmetrical uptake should be interpreted with some caution. The asymmetrical lesion in the right hip bone in Fig. 12b was clinically interpreted as bone metastasis by physicians.
- It is precisely that the symmetrical manifestation of hotspots in shoulder, elbow, hip, and knee joints, the arthritic image in Fig. 12c was incorrectly classified into the normal class. This further shows the difficulty of automated diagnosis of diseases with SPECT bone imaging. Non-oncological indications, however, are very common in bone scintigraphy including arthropathies and other bone injuries.

The experimental results for evaluation metrics demonstrate the automated classification performance by dSPIC compared to physicians’ manual diagnosis results that are served as ground truth in the LabelMe-based image annotation processing. The quantitative value for accuracy metric in Table 5 reveals that more than 77% of the testing samples have been correctly classified. The overall performance on distinguishing images between different classes reaches up to 78.6% (i.e., F-1 score = 0.7860). However, it is still a challenging task to make an accurate distinction between the normal and arthritic samples according to the evaluation metrics and confusion matrix depicted in Fig. 9. For all misclassified samples, we asked a nuclear medicine physician and an oncologist to check them one by one and analyze the reasons that cause misclassification. Figure 12 provides three typical examples of misclassified whole-body SPECT images, where lesions are manifested as the rectangle areas in the images. Now, we present the medical explanation from two experts as follows:

- The normal variants of radiopharmaceutical uptake can contribute to image misinterpretation. The higher concentration of activity in shoulder joints, pelvis, and knee joints in the normal image from a 76-year-old male patient as illustrated in Fig. 12a was incorrectly detected as an arthritic one. The main reason is that the accumulation of radiopharmaceutical in bone depends on local flow, extraction efficiency and degree of osteoblastic activity.

**Fig. 13:**
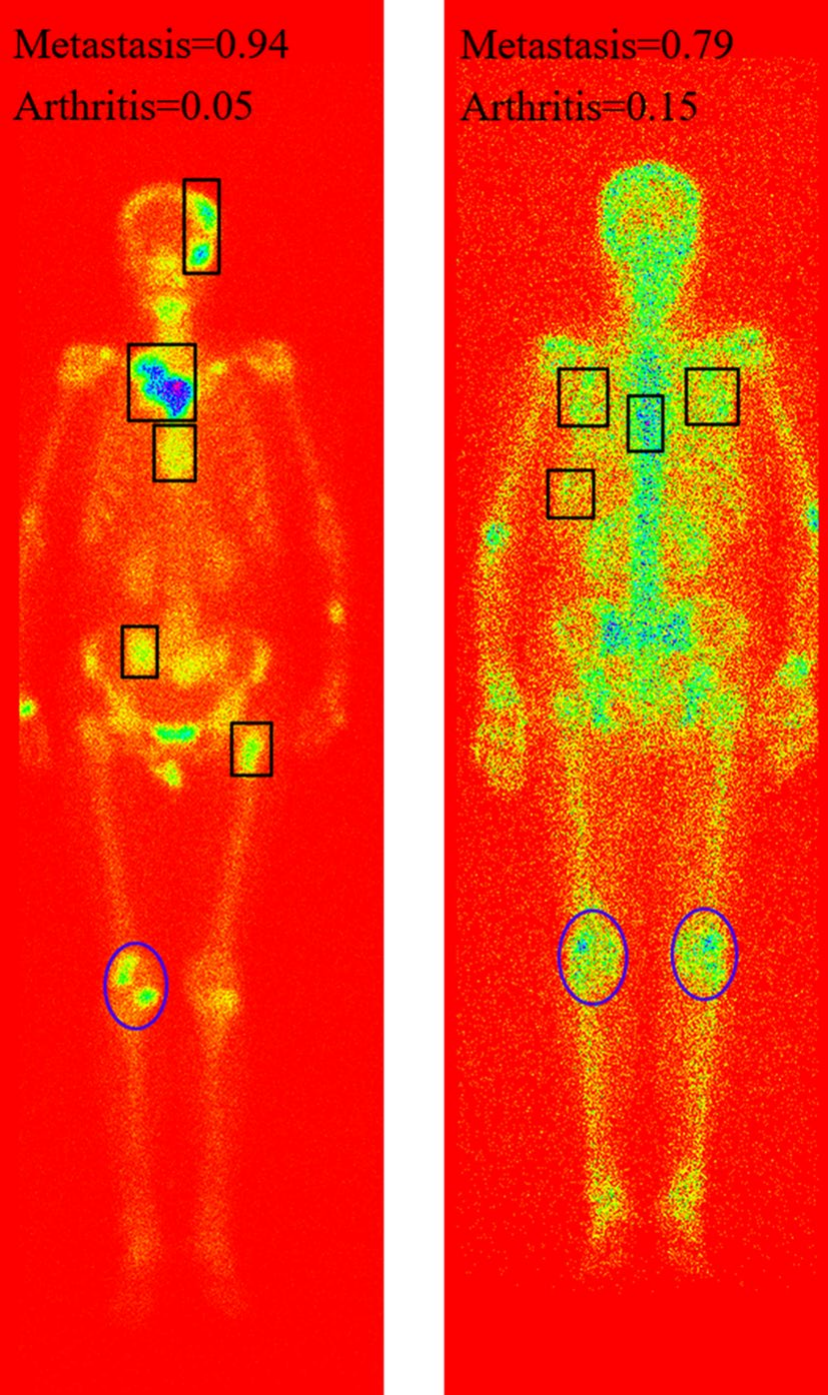
Examples of the metastasis and arthritis presenting in a single image simultaneously, with the metastatic and arthritic lesions delineating by rectangle and ellipse respectively

For low-resolution SPECT bone images, reliable automated diagnosis of diseases is still a challenging task. Especially, when multiple lesions of different diseases present in an image simultaneously, it will be more and more intractable to identify and distinguish them accurately. Figure 13 provides examples of occurrence of the metastatic and arthritic lesions in a single whole-body SPECT image. As a result, the Top-1 probability for bone metastasis is 0.94 and 0.79 in two images, and the Top-2 probability is 0.05 and 0.15 indicating arthritis.

We further examine the impact of pooling operation on classification performance by removing two pooling layers from our self-defined network dSPIC in Table 4. The experimental evaluation conducted on the testing samples in dataset D_2 obtains inferior performance (see Table 9), showing the necessity of pooling operation in CNN-based SPECT image classification with small-scale dataset. This is because pooling has the ability to eliminate overfitting by reducing the number of parameters as well as make the network invariant to geometric transformation (e.g., transformations, distortions and translations) in the input image.

**Table 9:** A comparison of the classification performance obtained by dSPIC-AM with and without pooling layers on the testing samples in dataset D_2

Classifer	Accuracy	Precision	Sensitivity	Specificity	F-1 score	AUC
dSPIC-AM with pooling	0.7747	0.7883	0.7863	0.8820	0.7860	0.9272
dSPIC-AM without pooling	0.7558	0.7561	0.7740	0.8642	0.7649	0.9060

In summary, the developed network dSPIC is workable for automated diagnose of diseases with whole-body SPECT bone scintigraphy images. However, we need to be clear that automated disease diagnosis with the low-resolution, large-size SPECT images is still in its infancy. More attention needs to be paid to improve the diagnosis accuracy and robustness from both the medical and technical fields, by following the potential lines of research below.

- With large-scale dataset of whole-body scintigraphic images, more representative features can be extracted from images for each kind of diseases by CNNs-based deep classification network. This would contribute to improving the ability of distinguishing between classes of concern.
- Statistical analysis conducted on large-scale data of scintigraphic images and pathologic findings would have the potential to develop a multi-modal fusion method, enabling higher performance for automated detection of diseases with whole-body bone scintigraphy.

## Conclusions

With SPECT imaging data collected from real-world clinical examinations, in this work, we have developed a CNN-based classification network, dSPIC, to automatically diagnose potential diseases without handcrafted features by physicians. The process of data preprocessing and data augmentation has been detailed. The built classification network has been elaborated. Experimental evaluation conducted on real-world SPECT images has been presented, achieving the best average of 0.7747, 0.7883, 0.7863, 0.8820, and 0.7860 for accuracy, precision, sensitivity, specificity, and F-1 score, respectively.

In the future, we plan to extend our work in the following directions.

First, we intend to collect more real-world SPECT images to comprehensively evaluate the developed deep network. Accordingly, optimization and improvement will be done for developing more robust, effective, and efficient diagnosis method.

Second, we attempt to develop an integrated classification method by introducing auxiliary information (e.g., geometric symmetry) into the network for more reliable diagnosis of various diseases with whole-body SPECT bone scans.

Last, we plan to develop other deep networks, exclusively targeting at the classification task of SPECT bone images for enlarging the current research domain of SPECT medical image analysis.

## Data Availability

The dataset is available only upon request by emailing Ms. Rong Wang (1160023677@qq.com) due to the ethical restrictions on sharing the de-identified data of SPECT bone scans. The Ethics Committee of Gansu Provincial Hospital has imposed ethical restrictions on the de-identified data because the SPECT bone scan images contain potentially sensitive information of patients.

## Abbreviations

SPECT: Single photon emission computed tomography
PET: Positron emission tomography
CT: Computed tomography
MRI: Magnetic resonance imaging
99mTc-MDP: 99MTc-methylene diphosphonate
CNN(s): Convolutional neural network(s)
GAN: Generative adversarial network
DCGAN: Deep convolutional generative adversarial network
ReLU: Rectified liner unit
ROC: Receiver operating characteristic
AUC: Area under the curve
YOLO: You only look once
DICOM: Digital imaging and communications in medicine
